# Aberrant Left Colic Artery and Its Surgical Implications

**DOI:** 10.7759/cureus.16397

**Published:** 2021-07-14

**Authors:** Anett Christiena, Nagaraj Kapil, Irfan Ansari, Saravanan PS, Naveen Kannan

**Affiliations:** 1 General Surgery, Meenakshi Medical College Hospital and Research Institute, Kanchipuram, IND; 2 Surgical Gastroenterology, Meenakshi Medical College Hospital and Research Institute, Kanchipuram, IND; 3 General Surgery, Meenakshi Medical College Hospital and Research Institute, Chennai, IND

**Keywords:** aberrant left colic artery, sma, splanchnic vascular anomaly, colonic resection, colonic conduit

## Abstract

Arterial anomalies of the viscera are not unusual. Of the arterial anomalies, the celiac and the superior mesenteric anomalies are well studied and reviewed in the literature. These variations are due to changes occurring during the development of vessels. Also, the variations in the colonic blood supply have been detailed in the context of conduit surgery in esophageal replacement and oncological resections. Of these, the rarer anomaly is the aberrant left colic artery (ab LCA). Previously described in various anatomic descriptions; it has never been reported in a clinical situation. A middle-aged female presented with abdominal pain and lower gastrointestinal (GI) bleed. On further evaluation, she was diagnosed to have transverse colon malignancy. She underwent extended right hemicolectomy with complete mesocolic excision and D3 lymphadenectomy as classically described. During the dissection, she was found to have an LCA arising from the superior mesenteric artery (SMA) just below the inferior border of the pancreas two centimeters higher to the origin of the middle colic artery. This artery was carefully dissected and preserved. Injury of the ab LCA is possible given the unusual course of the artery. Implications of iatrogenic injury in colonic and pancreatic surgeries may result in additional morbidity which is discussed in detail.

## Introduction

Normal mesenteric circulation relies on three major arterial trunks which are persistent intersegmental arteries that fail to regress. Celiac, superior mesenteric artery (SMA), and inferior mesenteric artery (IMA) are the three major arterial trunks that supply the foregut, midgut, and hindgut respectively. In the past anatomical studies contributed to the knowledge of their aberrations but with time, high-resolution imaging including contrast-enhanced CT has contributed to the knowledge of arterial anomalies [1]. They have become instrumental in planning interventional radiological and surgical procedures.

Very few studies have surfaced on the aberrant left colic artery (ab LCA). The IMA after giving rise to LCA then gives multiple sigmoidal branches and continues as a superior rectal artery. The LCA after its origin divides into ascending and descending branches. The ascending branch forms the marginal artery and supplies the left one-third of the transverse colon, splenic flexure, and the variable extent of descending colon. Aberrations in the origin of the left colic artery is an unusual phenomenon only reported in anatomical studies.

## Case presentation

**Figure 1:**
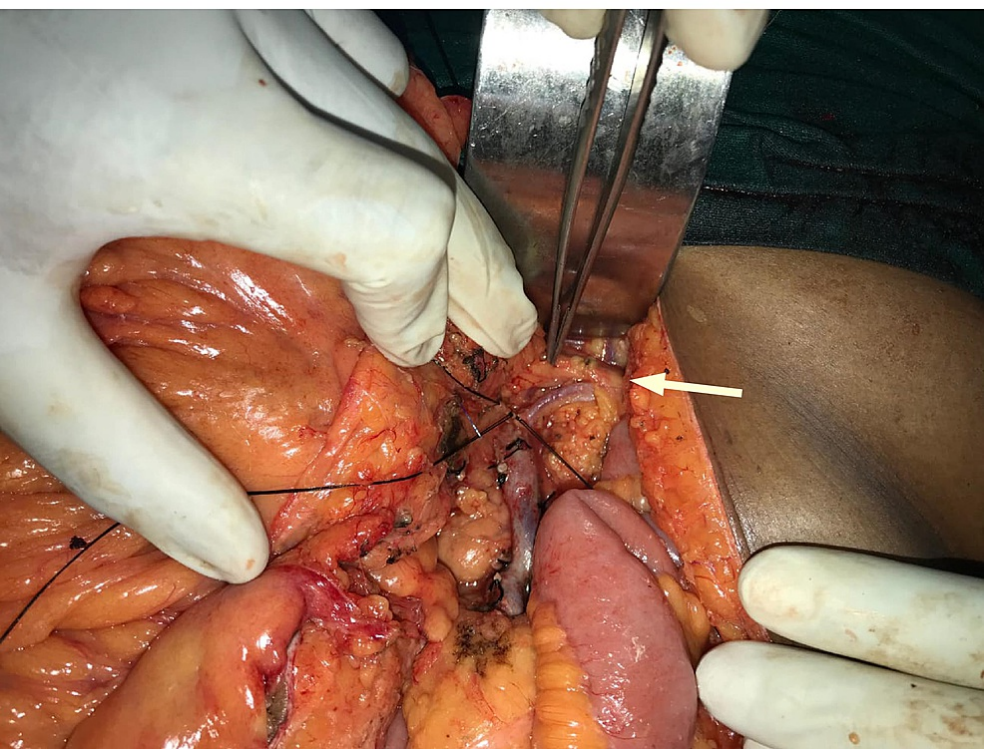
Intra-operative picture of ab LCA. Intra-operative picture of the ab LCA with an arrow pointing towards it ab LCA, aberrant left colic artery

**Figure 2:**
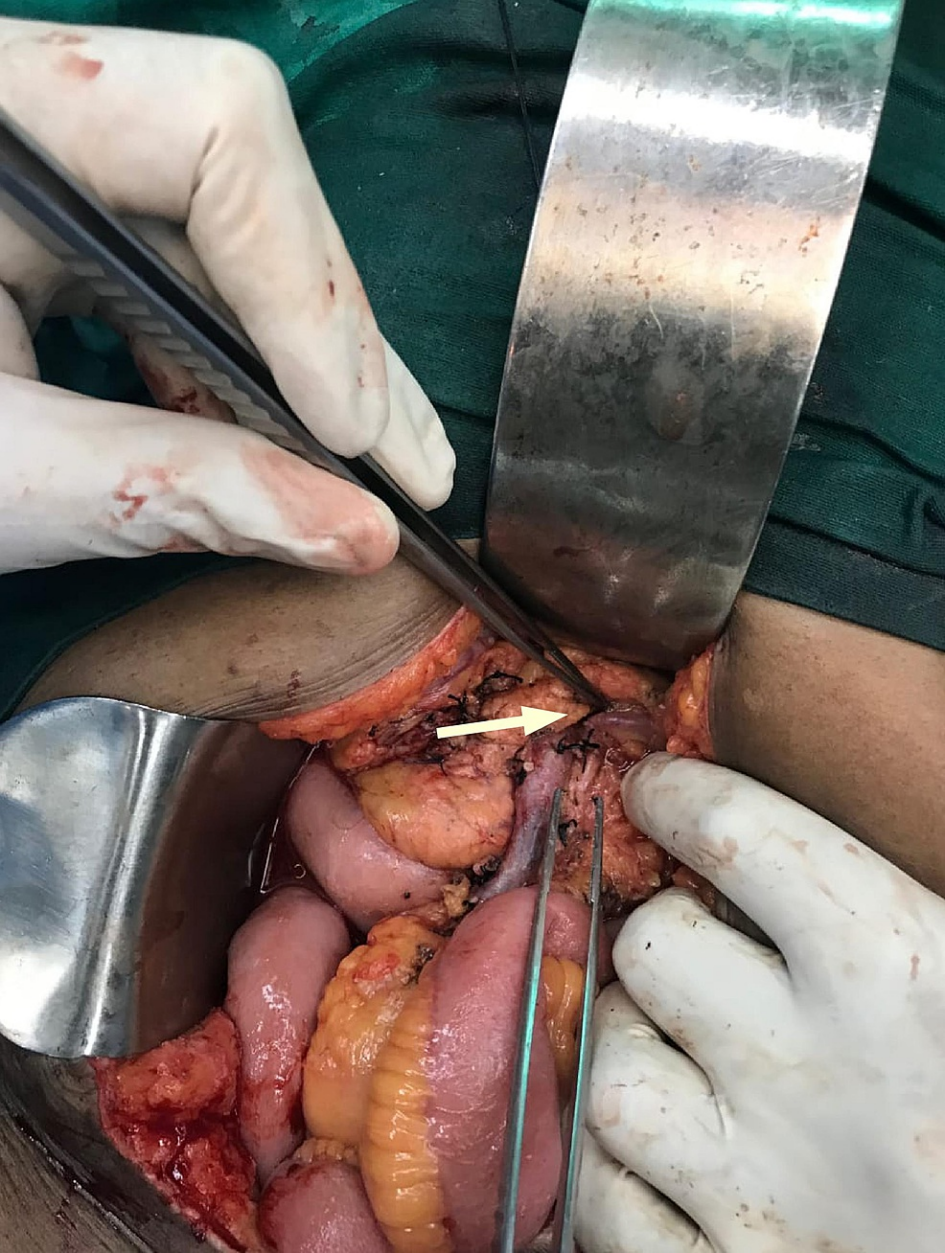
Intra-operative picture of ab LCA. Intra-operative picture of the ab LCA with an arrow pointing towards it ab LCA, aberrant left colic artery

A middle-aged female presented with abdominal pain and lower gastrointestinal (GI) bleed for two months. She also had a significant loss of weight. She did not have any vomiting, abdominal distention, and jaundice. Her general condition was preserved with an Eastern Cooperative Oncology Group (ECOG) performance status score of one. Abdominal examination revealed a tender vague mass in the right upper quadrant. She underwent colonoscopy after bowel preparation which showed a circumferential ulceroproliferative lesion in the proximal transverse colon. Biopsies were taken from the lesion which was reported as well-differentiated adenocarcinoma. Further dual phase-contrast CT was done which showed asymmetric irregular wall thickening in the right one-third of the transverse colon with minimal pericolic fat stranding. She was optimized and taken up for surgery. She underwent open extended right hemicolectomy with complete mesocolic excision and D3 lymphadenectomy as classically described [2]. She underwent extended right hemicolectomy with D3 lymphadenectomy in view of the location of the tumor in the mid transverse colon and the presence of enlarged infrapyloric nodes, respectively. During the procedure, an unusually large-caliber artery could be traced along the inferior border of the pancreas. Further dissection of the artery revealed that the origin was from the SMA just inferior to the pancreas about two centimeters above the origin of the middle colic artery (Figure 1). Also, it is divided into two branches immediately after origin (Figure 2). The right branch traveled into the right transverse mesocolon and the left branch course was into the mesocolon of the left transverse colon and splenic flexure almost similar to the middle colic artery. Pulsations of the left branch could be traced to the margin of the descending colon. The right branch was taken down and the left branch was preserved. Ab LCA branching pattern was contrary to the description in the literature previously [1].

**Figure 3:**
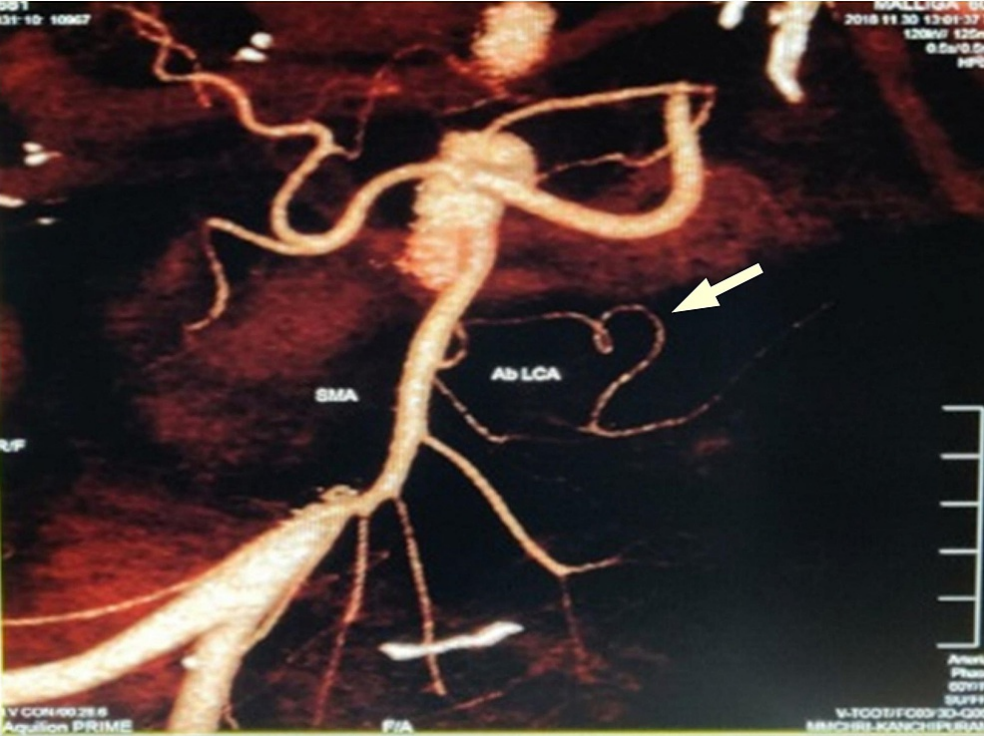
CT angiogram denoting ab LCA. CT angiogram of abdomen denoting ab LCA with an arrow pointing towards it ab LCA, aberrant left colic artery

**Figure 4:**
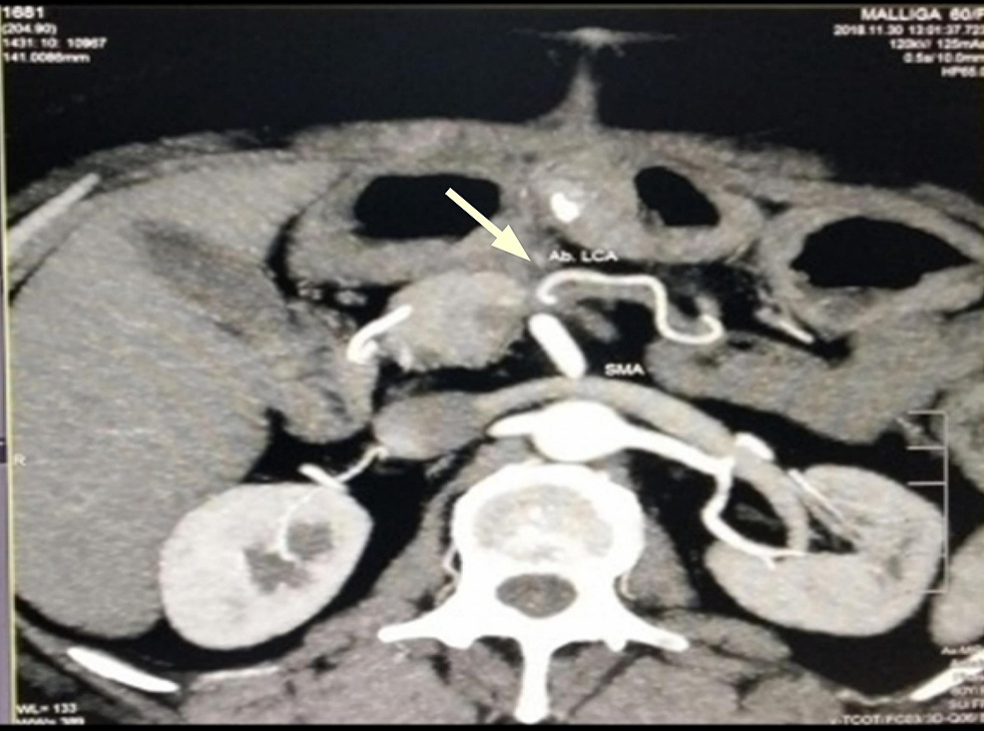
Contrast CT abdomen of ab LCA - coronal section. Contrast CT abdomen of ab LCA with an arrow pointing towards it ab LCA, aberrant left colic artery

**Figure 5:**
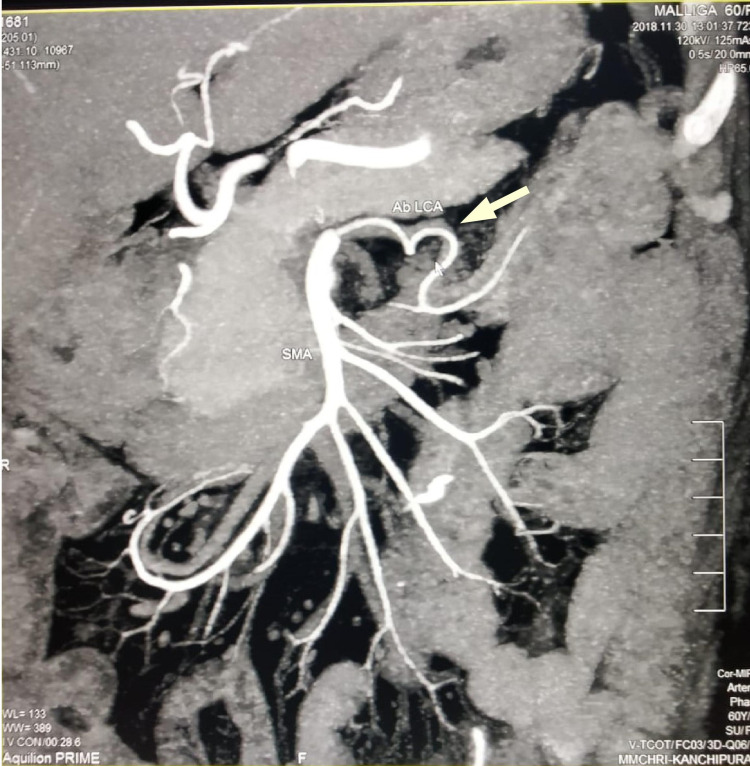
Contrast CT abdomen of ab LCA - sagittal section. Contrast CT abdomen of ab LCA - sagittal section with an arrow pointing towards it ab LCA, aberrant left colic artery

The report was a well-differentiated adenocarcinoma involving muscularis propria with all 32 lymph nodes negative for tumor deposits and was staged as pT2N0. As there were no high-risk features, she was considered for surveillance. Follow-up imaging -- triple-phase contrast-enhanced CT was done at one year and herewith the CT reconstruction images are displayed which clearly showed the aberrant origin of the LCA and its course (Figures 3-5).

## Discussion

Definitive digestive arterial trunks (the celiac trunk, the SMA, and the IMA) arise from several primitive arteries which accounts for some arteries induced by a craniocaudal shift along the aortic tube.

Aberrant LCA is very rare and previous descriptions are based on anatomical studies only [3-8]. This is the first clinical case report in the literature that has detailed the unusual origin, course, and branching.

According to the study done by Amonoo-Kuofi on a cadaver, a variant vascular pattern was identified, i.e. an accessory right hepatic artery arising from SMA, a middle colic artery from splenic artery and an accessory left colic artery from SMA [4]. Similarly, in a cadaver a left aberrant accessory colic artery was identified by Rusu et al., which divided into two branches, ascending anastomosed with middle colic artery and descending with proper left colic artery [5]. Likewise, during cadaveric dissection Mishra et al. have observed an ab LCA arising four centimeters distal to the origin of SMA, which crossed the aorta from right to left giving two branches, ascending and descending respectively [6]. In a case report by Nayak et al. LCA was identified arising from SMA giving two branches right and left, and the right branch traverses along the inferior border of the pancreas and crosses the left kidney to supply left colic flexure and descending colon, which is in close relation with pancreas and is vulnerable to get injured during pancreatic surgery [7]. In contrary to all these scenarios, in our case, the LCA originated from the SMA just inferior to the pancreas about two centimeters above the origin of the middle colic artery. In the presence of a left accessory aberrant colic artery, the proper left colic artery is absent, atrophic, or displaced. Likewise in our case, proper LCA supposed to be arising from IMA is absent.

Randomized controlled trial clearly demonstrates the necessity of 3D reconstruction imaging of arteries to avoid dilemmas during surgery, thereby shortening the operative time [9]. The volume-rendered 3D CT imaging of artery before surgery will help us to know the aberrant arteries or accessory arteries arising from the celiac trunk, SMA and IMA, which will be useful for surgical planning. As in our case, LCA was arising from the SMA instead of IMA and it is inevitable and safe to do imaging before surgery, as there are more chances of missing out the aberrant artery on the table and its ligation will lead to non-viability of that segment of the colon which can result in complication postoperatively and the need for resection of that particular segment of colon. Henceforth variation in the vascular anatomy can affect the surgical intervention.

Apart from the anatomical description, further understanding of the implications of the said aberrant artery is discussed herewith.

Surgical implications

Oncological resections in the colon are solely based on the principal artery supplying that segment of the colon. In case of aberrations such as in our case, routine left hemicolectomy for left colon cancer would have become inadequate due to the failure to dissect and divide the LCA at its origin [1, 10-12]. In our own case, injury of the aberrant LCA during dissection would have been disastrous, and extended colonic resection to viable segment, sometimes subtotal colectomy would have been required.

As described by Nakao, the middle colic artery is usually divided to expose the superior mesenteric vein (SMV)-SMA at the infrapancreatic region [13]. In this approach, an assumption is made as to the viability of the colon being maintained by the ileocolic artery, left colic artery, and a continuous marginal artery of Drummond. With this aberrant course of the LCA, injury or ligation at the SMA origin would have made colon non-viable for variable segments and demanded a combined colonic resection further adding to the morbidity.

In colonic conduit surgery, generally, the left colonic supply is considered more robust and consistent. Hence, the colonic conduit is based on LCA and transverse colon being used for replacement of esophagus and a routine division of middle colic after trial clamping is done. Inability to recognize the aberrant origin of the LCA may result in inadvertent injury thereby leaving the colon as a non-viable option for esophageal reconstruction.

Pseudoaneurysms involving the colic artery are rarely reported [14]. Infrapancreatic course along the surface of the pancreas may predispose this aberrant colic artery to pseudoaneurysm formation. The unusual site and territory of the pseudoaneurysmal bleed should prompt a search for anatomic aberrations.

## Conclusions

This is the first clinical case report describing the origin and the course of this rare, ab LCA. Given the unusual origin and course, this artery is prone to iatrogenic injury. Surgical implications of such an arterial aberration and its injury in colonic resections, colonic conduit surgery, and pancreatoduodenectomy are discussed.
